# Impacts of COVID-19 on the oil and gas industry in Brazil

**DOI:** 10.47626/1679-4435-2022-1015

**Published:** 2023-11-24

**Authors:** Brenda do Amaral Almeida, Camila Henriques Nunes, Eliana Napoleão Cozendey-Silva, Marcelo Juvenal Vasco, Liliane Reis Teixeira

**Affiliations:** 1 Programa de Pós-Graduação em Saúde Pública e Meio Ambiente, Escola Nacional de Saúde Pública Sergio Arouca, Fundação Oswaldo Cruz (Fiocruz), Rio de Janeiro, RJ, Brazil; 2 Instituto Federal Fluminense, Campos dos Goytacazes, RJ, Brazil; 3 Centro de Estudos da Saúde do Trabalhador e Ecologia Humana (CESTEH), Fiocruz, Rio de Janeiro, RJ, Brazil; 4 Federação Nacional dos Petroleiro, Rio de Janeiro, RJ, Brazil; 5 Sindicato dos Petroleiros do Litoral Paulista, Santos, SP, Brazil

**Keywords:** occupational health, oil and gas industry, public health, COVID-19, saúde do trabalhador, indústria de petróleo e gás, saúde pública, COVID-19

## Abstract

This study aimed to analyze the progression of COVID-19 cases on oil and gas workers in Brazil. This is a descriptive research based on secondary data available in the COVID-19 Monitoring Bulletins of the Ministry of Mines and Energy and news about outbreaks on digital media. The findings show that the numerous news reports published on digital media indicate unhealthy working conditions, and workers’ representatives appear as the main source of these findings. Managers’ failure in health surveillance of this working class were also observed as to the frequent omission of official data and compulsory notifications on the health and safety of oil and gas workers.

## Abstract (pt)

Este estudo teve como objetivo analisar a evolução dos casos de trabalhadores do setor de petróleo e gás afetados pela covid-19 no Brasil. Tratou-se de uma pesquisa descritiva, realizada a partir de dados secundários disponíveis nos boletins de monitoramento da covid-19 do Ministério de Minas e Energia e das notícias sobre surtos de covid-19 em petroleiros publicadas nas mídias digitais. Concluiu-se que as inúmeras notícias publicadas pelas mídias digitais refletem condições de trabalho insalubres, e os representantes dos trabalhadores surgem como a principal fonte desses achados. Foram observadas ainda falhas na vigilância em saúde por parte dos gestores dessa classe trabalhadora no que concerne à frequente omissão de dados oficiais e às notificações compulsórias sobre a saúde e a segurança dos petroleiros.

## INTRODUCTION

The first outbreak of COVID-19, a disease caused by SARS-CoV-2, broke out in the city of Wuhan, China, in December 2019, and on March 11, 2020, the World Health Organization (WHO) declared it a pandemic.^1^

According to a Johns Hopkins University (JHU) epidemiological bulletin, 499,167,244 cases and 6,180,051 deaths due to COVID-19 have been confirmed worldwide as of April 11, 2022. The United States was the country with the highest number of total cases (80,410,247), followed by India (43,036,132), Brazil (30,153,979), France (27,136,925), United Kingdom (21,807,354), Russia (17,745,453), Turkey (14,965,867), and Spain (11,627,487).^2^

As a result of difficulties in accessing Brazil healthcare services, limitations in identifying and diagnosing cases, and shortage of human resources, it is likely that many cases have remained underreported. Therefore, it is possible that the magnitude of the pandemic is greater than statistically reported.^3^

Importantly, the constant circulation of workers in the various production chains has contributed to spreading the virus, demonstrating the pivotal role of the world of work in the organization and functioning of societies and populations.^4^

Some researchers are convinced that COVID-19 should be considered a new work-related disease, as it affects people who leave their homes to work and thus remain continuously at risk of interpersonal contact and potentially contaminated surfaces in the workplace, pointing to the relevance of infection control in the workplace to protect workers’ health.^5-8^

In the context of the COVID-19 pandemic, considering the multiple occurrences of cases in workplaces, an outbreak occurs when there are two or more test-confirmed cases, among individuals associated with a specific non-household environment, with disease onset dates within 14 days.^9^

Legislative Decree No. 10,282/2020 regulated the essential activities and services of workers in Brazil, including the oil and gas industry (“oil production and production, distribution, and marketing of fuels, biofuels, liquefied petroleum gas, and other petroleum products”) as an essential activity that must remain in operation during the COVID-19 pandemic.^10^

As a result, international bodies have published several recommendations to tackle COVID-19. However, gaps are still found in the monitoring of workers’ health and safety measures, sometimes analyzed entirely apart from working conditions. These conditions are not always of managers’ concern, who often do not take into account training and personal protective equipment (PPE) as important (although not sufficient) factors for workers, when they are even provided in insufficient quantity and quality for their activities.^11^

Thus, this study aimed to analyze the progression of COVID-19 cases on oil and gas workers in Brazil (from April 2020 to April 2021) as reported on digital media. We also sought to identify the main channels for news reports on outbreaks of the disease among oil and gas workers and compare them with data from official bodies.

## METHODS

This study was predominantly qualitative. For the analytical path of textual artifacts, Minayo’s content analysis proposal was assumed.^12^ Descriptive statistics were used to organize and represent the observed data, in order to help describe the progression of COVID-19 cases on oil and gas workers in Brazil.

Secondary data from the COVID-19 monitoring bulletins (number of confirmed and quarantined cases, hospital admissions, and deaths due to COVID-19) available on the Ministry of Mines and Energy (MME) website from April 2020 to April 2021 were used. According to a survey on reports (bulletin no. 1 to no. 52), the following findings were found: 11,427 cases were deemed as “confirmed and quarantined;” 535 “hospital admissions;” and 268 “deaths.”

Concurrently, news reports on digital media were searched on Google and analyzed using the terms: “surto covid 19 petroleiros” (COVID-19 outbreak oil and gas workers) and “surto covid 19 Petrobrás” (COVID-19 outbreak Petrobras), as well as news on outbreaks among oil and gas workers published on the Biblioteca Nacional Digital (BND, Brazilian ISBN agency), PressReader, and the websites of trade unions and industries linked to the Federação Nacional dos Petroleiros (FNP) and Federação Única dos Petroleiros (FUP) in the same period (April 2020 to April 2021).

The data found on digital media were organized in a table containing the following information: source, date, title, and summary of the news. After systematizing the news, information on outbreaks and cases of COVID-19 among workers and the number of workers diagnosed with COVID-19 (according to news reports) were collected.

The analysis and interpretation of the significant constructs^12^ about the COVID-19 cases on workers on oil and gas industry were performed as registration units were selected.

The analysis of secondary quantitative data from the MME comprised descriptive statistics on the information related to reported cases.

It is worth noting the challenge of obtaining reliable information on the number of workers diagnosed with COVID-19, as the MME constantly changes the data.

It is justifiable to search for news during the first 12 months of the pandemic because oil and gas workers were unable to interrupt their work activities to remain in isolation since the beginning of the pandemic, as enacted by Law No. 13,979, of February 6, 2020, which regulated the continuity of public services and essential activities in the country.^10^

Consequently, all labor activities involving oil production and distribution and marketing of fuels, biofuels, liquefied petroleum gas, and other petroleum products remained in operation, causing great impact on the health and safety of several workers on oil and gas industry.

As for ethical issues, this study used exclusively data in the public domain. Therefore, this study was not subjected to a Research Ethics Committee review according to Resolution 510/16 of the Conselho Nacional de Saúde (National Health Council).

## RESULTS

Specifically in Brazil largest oil and gas company, the gap between outsourced and permanent employees is remarkable. According to Petrobras’ 2020 sustainability report, the number of employees working in the oil and gas plants in 2020 was 41,885, while the number of outsourced employees was 91,426, and the Southeast and Northeast regions had more outsourced employees, respectively.^13^

Any information related to outsourced workers, such as work schedules, training and capacity building, for example, is the sole responsibility of their employers.

It is worth noting that, in this type of industry, workers perform occupational activities in risky, dangerous, complex, collective, continuous and confined environments.^14^

### INFORMATION PROVIDED BY THE MME

The first MME COVID-19 monitoring bulletin was released on April 20, 2020, approximately 1 month after the WHO declared the outbreak of COVID-19. According to this bulletin, in this period, 236 employees were confirmed with COVID-19 and 1,152 were suspected.^15^ On that same day, according to the Ministério da Saúde (MS, Ministry of Health), Brazil had 40,581 confirmed cases, 2,575 deaths, and a case-fatality rate of 6.3%.

**Figure 1:**
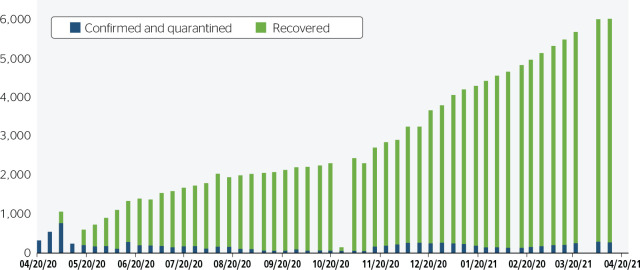
Confirmed and quarantined COVID-19 cases among oil and gas workers, between April 2020 and April 2021, according to official data of the Ministry of Mines and Energy.

**Figure 2:**
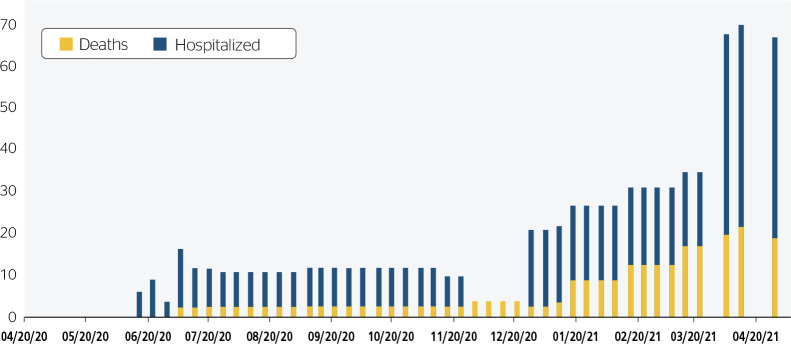
Cases of admission and death due to COVID-19, between April 2020 and April 2021, according to the Ministry of Mines and Energy official data.

The analysis of the data the MME released from April 2020 to April 2021 (Figures 1 and 2) shows an increasing number of cases and a history of changes in the reporting of official data, favoring misinterpretations on the actual health scenario of workers in the oil and gas industry, as well as the invisibility of outsourced oil workers.

The first two bulletins considered a total of 46,416 civil servants (excluding outsourced workers) and presented only data on suspected and confirmed cases of COVID-19, not reporting how many of them were quarantined. In the third bulletin released (05/04/2020), information on the number of recovered workers (231) was included, and a total of 151,539 workers were included (civil servants, outsourced employees, and contractors from other companies).^15^

Importantly, during the period studied, how some information was released in the bulletins changed. For instance, in the following week (05/11/2020), the MME again considered 46,416 civil servants (total of employees, a number that did not change even after voluntary termination, dismissals, and retirements), did not use the term “quarantine,” and presented only confirmed cases.^15^ Thus, figured dropped significantly in a week, since outsourced employees were no longer included. From this change in reporting, the number of confirmed cases dropped from 806 to 222 infected employees. The number of suspected cases decreased from 1,642 to 474 cases.

MME reports changed again in May, no longer reporting the number of suspected cases. As of suspected cases, the last report with 474 cases was released on May 11.^15^

Information on hospitalization of infected employees were initially released in bulletin 9 of the MME (06/15/2020)^15^ (Figure 2). According to bulletin 9, five workers were hospitalized, an insignificant number compared to the total number of confirmed and suspected cases reported until then. This may be a clear example of lack of actual information about the health impacts oil and gas workers have experienced.

The number of hospitalized workers (17 cases) remained the same from 12/28/2020 to 03/22/2021, with a sharp increase in the bulletin issued on 03/29/2021, from 17 to 47 cases.^15^

Fatalities were only reported on July 6, 2020 (bulletin 12). Then, three deaths were reported, and this number remained until January 11, 2021 (bulletin 39), when four deaths were reported. From January 18 to February 8, 2021 (bulletins 40 to 43), the number of deaths remained stable at nine deaths. Since then, death reports have changed, differentiating the deaths of oil and gas workers on vacation, working remotely, and on site.^15^

As of 09/14/2020, the total number of COVID-19 cases in Petrobras was equivalent to 4,448.9 cases/100,000 inhabitants, which is more than twofold (2.15) the incidence recorded in Brazil (2,067.9), according to information provided by the Ministry of Health’s Coronavirus Brazil Panel.^16^

However, only three cases were reported in MME bulletin No. 25 of 10/05/2020, proving inconsistent with the official data Petrobras had submitted to the MME. This was presumably due to omission of deaths of outsourced workers, and certainly because not all deaths of civil servants were reported.

This analysis was carried out about six months later, on 03/29/2021, and the incidence of COVID-19 cases in the state-owned company was 12,700.4 cases/100,000 inhabitants, which corresponds to more than a twofold increase compared to the country average (5,983.3 cases/100,000 inhabitants) on the same date. As of 04/12/2021, 5,749 Petrobras civil servants had been diagnosed with COVID-19.^17^

Especially for outsourced employees, the situation was even more serious considering the deaths of Petrobras civil servants (4) and outsourced employees (15), having reported a total of 19 deaths due to COVID-19 by August 2020, surpassing the number of deaths due to occupational accidents between 2004 and 2016.^18,19^

### NEWS ABOUT COVID-19 OUTBREAKS IN THERMOELECTRIC PLANTS, REFINERIES, AND OIL RIGS REPORTED ON DIGITAL MEDIA

When searching through digital media, it was observed that most of the news published in newspapers has as a source the unions of oil and gas workers, that is, most of the news from unions was repeated in newspapers of great distribution.

It is known that the underreporting of cases is a sad reality in Brazil. Therefore, in many cases, the information comes from family members who seek help from unions, coworkers, social media of those workers, among others.

**Figure 3:**
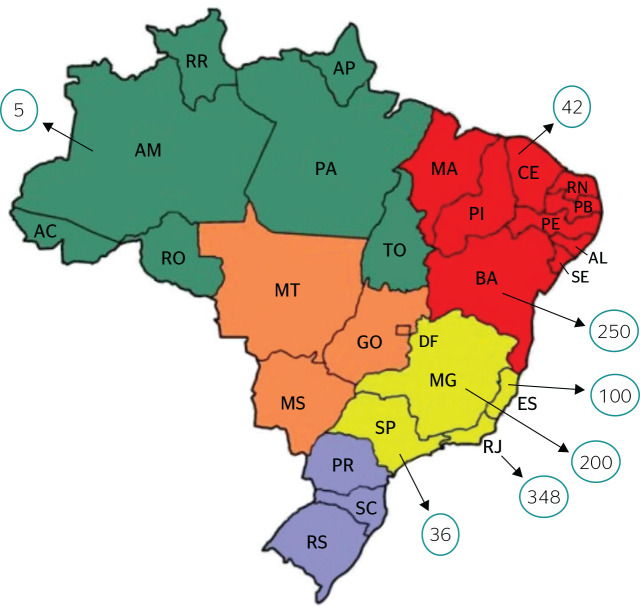
COVID-19 cases on oil and gas workers in Brazil. Source: Elaborated by the authors based on news released on digital media. State of Bahia (BA): 224 cases at Refinaria Landulpho Alves Mataripe and 26 cases on a Transpetro tanker, totaling 250 cases; State of Ceará (CE): 42 cases on rigs PXA-1 and PXA-2; State of Espírito Santo (ES): 61 cases on rig FPSO Capixaba, 34 cases on SBM-ES and 5 cases on P-58, totaling 100 cases; State of Minas Gerais (MG): 200 cases at Refinaria Gabriel Passos (Regap); State of Rio de Janeiro (RJ): 112 cases in Campos Basin (exact location not stated), 22 cases on P-12, 5 cases on P-56, 31 cases on P-69, 8 cases on P-18, 1 case on P-75, 22 cases on P-56 and P-25, 5 cases on P-47, 29 cases on P-74, 4 cases on P-75, 10 cases on P-48, 48 cases on P-54, 8 cases on P-31 and 5 cases on P-38, totaling 348 cases; State of São Paulo (SP): 5 cases on rig Merluza, 6 cases on Mexilhão, 8 cases on P-70, 6 cases on P-69, and 11 cases at Refinaria Presidente Bernardes - Cubatão (RPBC), totaling 36 cases; State of Amazonas (AM): 5 cases in Urucu.

From April 2020 to April 2021, 67 news reports on COVID-19 cases were identified on different digital media, including 50 news reports on trade unions websites as the main source of information. After analyzing the textual elements of these news reports, 35 were identified as addressing COVID-19 outbreaks on oil rigs and refineries across states, as shown in Figure 3.

Some news reports found on trade unions websites contained a description of the number of cases of COVID-19 in different work settings, especially on oil rigs, and these numbers were calculated per state (Figure 3).

We observed that, considering a total of 35 reported outbreaks, 981 workers were infected with COVID-19, mostly in Southeastern Brazil.

**Figure 4:**
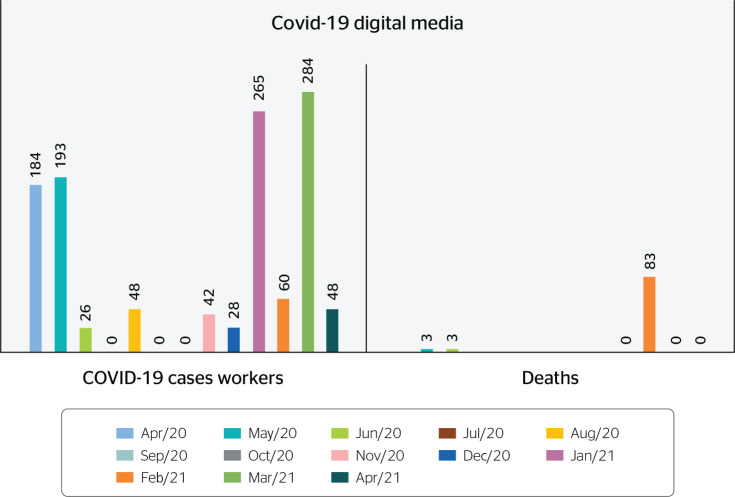
Confirmed cases of COVID-19 and deaths of oil and gas workers.

Importantly, the primary source of information for these reports was the websites of representatives of the trade unions. The high number of deaths was first reported in the news in February 2021, as described in Figure 4.

### TRADE UNIONS AS A SOURCE OF INFORMATION

Due to the COVID-19 pandemic since March 2020, oil and gas industry has implemented teleworking to reduce clerical work. However, health protection measures have been neglected for workers in operational facilities, especially offshore oil and gas facilities, according to trade union representatives.

The consolidated information provided by the FNP and the FUP is relevant and can be considered a useful source to track cases and deaths due to COVID-19 in the Petrobras System.

Together with trade union representatives, the FUP has acted directly in holding hearings with Petrobras, mediated by prosecutors from the Public Ministry of Labor (MPT) and auditors from the Regional Superintendence of Labor and Employment (SRTE), linked to the Ministry of Economy, to demand improvements to workers’ healthcare in the pandemic.

Failure to communicate is a major cause of workers’ dissatisfaction with the measures the companies have implemented. There are many questions about harmful management to health and safety resulting from companies’ decisions, such as increasing working hours and boarding time, as well as the hiring of new outsourced workers and failure to issue a work accident report (CAT) in cases of deaths due to COVID-19.

The meetings between trade union representatives and the companies operating on the oil and gas industry are attempts to address several relevant guidelines related to COVID-19: failure to issue a CAT in case a worker is infected with SARS-CoV-2 at work or while commuting; outbreak reports on rigs; failure to include outsourced workers in weekly epidemiological bulletins; introduction of new work schedules in disagreement with the Collective Bargaining Agreement; underreporting cases and deaths; failure to provide adequate testing and prevention measures for all civil servants and outsourced workers; among others.

In this scenario of countless challenges, trade unions on the oil and gas industry play an important role in sharing information to workers and society as well. We observed that a significant number of news reports on COVID-19 outbreaks reported on digital media and major newspapers rely on trade unions as their sources of information.

In Brazil, 50 news reports on COVID-19 outbreaks were found on websites of oil and gas unions during the study (Tables 1 and 2).

**Table 1:** Reports on COVID-19 outbreaks on oil and gas workers (April to December 2020)

Month/2020	Trade Unions	n
April	Sindipetro do Litoral Paulista	2
	Federação Única dos Petroleiros	1
May	Federação Única dos Petroleiros	1
	Sindipetro do Litoral Paulista	1
June	Sindipetro do Norte Fluminense	2
	Sindipetro do Litoral Paulista	1
July	Sindipetro de São Paulo	1
	Federação Nacional dos Petroleiros	1
August	Federação Única dos Petroleiros	3
	Federação Nacional dos Petroleiros	1
	Sindipetro do Rio de Janeiro	1
September	Sindipetro de Caxias	1
October	Federação Nacional dos Petroleiros	1
	Federação Única dos Petroleiros	1
November	Federação Única dos Petroleiros	2
	Sindipetro da Bahia	2
	Sindipetro do Litoral Paulista	1
December	Sindipetro do Litoral Paulista	2
	Federação Única dos Petroleiros	1
	Sindipetro do Espírito Santo	1
	Sindipetro de São Paulo	1
Total		28

Sindipetro = Sindicato dos Petroleiros (oil and gas trade union).

**Table 2:** Reports on COVID-19 outbreaks on oil and gas workers (January to April 2021)

Month/2020	Trade Unions	n
January	Sindipetro do Litoral Paulista	1
	Sindipetro de São Paulo	1
	Sindipetro do Espírito Santo	1
	Federação Única dos Petroleiros	2
	Sindipetro da Bahia	2
February	Sindipetro do Rio de Janeiro	3
	Central Única de Trabalhadores	2
	Sindipetro da Bahia	1
March	Sindipetro do Rio de Janeiro	1
	Sindipetro do Litoral Paulista	1
	Federação Única dos Petroleiros	1
April	Sindipetro do Rio de Janeiro	1
	Federação Única dos Petroleiros	2
	Federação Nacional dos Petroleiros	1
	Sindipetro de Minas Gerais	1
	Sindipetro do Norte-fluminense	1
Total		22

Sindipetro = Sindicato dos Petroleiros (oil and gas trade union).

According to data released by the Sindicato dos Petroleiros (Sindipetro) do Norte Fluminense, at least 15 facilities (Petrobras [13], Perenco [1], and Trident [1]) were denounced, totaling 1,900 civil servants and outsourced workers, with 129 confirmed cases of COVID-19 (17% of workforce) and 327 on sick leave.^19^

According to information from trade unions and other insiders linked to unions, the number of deaths at the FUP was 60: 12 civil servants and 48 outsourced workers.

The following data on deaths were found on a search for news on digital media of trade unions: 1 death in onshore fields in Bahia, 1 death in Espírito Santo, 2 deaths in Campos Basin (Rio de Janeiro), 1 death at Transpetro, 4 deaths at Reduc, 5 deaths at Complexo Petroquímico do Rio de Janeiro (COMPERJ), totaling 12 deaths in Rio de Janeiro; 3 deaths in Cubatão, São Paulo; 1 death in Urucu and 1 death at Transpetro, totaling 2 deaths in Amazonas. This amounts to 19 deaths from April 2020 to April 2021.^19^

## ANALYSIS AND DISCUSSION

The essential role of public health is to take actions aimed at the population at greatest risk of becoming ill to face the pandemic, such as oil and gas workers who are considered essential service workers. However, a detailed analysis is necessary to understand how illness occurs and thus determine prevention for this population.^20,21^

This study found a significant number of infected and hospitalized oil and gas workers. However, some groups of workers are known not to have made a specific notification of COVID-19 cases; thus, there is a growing underreported number of infected individuals, making it difficult to assess the place and circumstances of dissemination, undisaggregated indicators in statistics, in addition to failure to identify the foci of virus dissemination related to occupational activities.^21^

The fragility of labor contractualization and damage to employment have made many workers vulnerable in terms of rights.

The current measures to tackle the pandemic mirror the current model of the economic system, in which social distancing and prolonged and restricted quarantine are used to suppress outbreaks. As a result, workers remain working and circulating, consequently exposed to contamination, illness, and death.^17^

Studies have found that work-related COVID-19 cases result from insufficient, inadequate, or no health and safety measures against SARS-CoV-2 in workplaces and processes. Inadequate measures include the following: failure to adopt distancing between workers, inadequate or no PPE, poor working conditions, inadequate disinfection of work facilities and equipment, exhausting working hours, high demands (some workers have had their duties increased), no training, and no administrative measures to rearrange work processes.^22,23^

Workplaces and processes play a crucial role in the rapid spread of the virus, especially when confined, in unventilated environments, with air-conditioning systems, and difficulties to completely ventilate the room, which favors direct or indirect transmission and infection with COVID-19.^24^

Some facilities, even if they are not health facilities with a high or very high risk of contamination, favor the spread of the virus. Thus, they are major sources of biological risk for SARS-CoV-2, such as confined ship and oil rig settings, with crowds in closed spaces, inadequate ventilation, and sharing of workstations and equipment. In these places, the contamination rate of 1 case of COVID-19 can evolve to 16 new cases, while in a safe community and on land, the contamination of 1 case of COVID-19 can reach up to 4 new cases of the disease.^23^

In addition to the biological risk in workplaces on the oil and gas industry, workers have faced several changes, including work schedules, with previous 7-day isolation in a hotel before boarding, 21 days on board and 14 days off to reduce the number of Petrobras civil servants in offshore facilities, excluding outsourced workers.^25^

However, the increased exposure of these workers to various psychosocial factors should be considered, including changes in the working regime and schedule associated with pressure and precarious conditions imposed on workers, resulting in greater physical exhaustion and psychological disorders.

The current government has made several changes to occupational safety regulations, most of which trade unions and the MPT have criticized and employers have supported. These changes aim to reduce demands on businesses to make occupational health poorer, thus setting back labor achievements.^26^

Although the data released to the MME on COVID-19 infections among workers have been continuously updated, they do not accurately portray the reality of oil and gas workers, both in cases of death and number of infected workers. According to Petrobras 2020 sustainability report, discrepancies have been found in data presented to the MME as to the total number of civil servants, for example.

The first MME bulletin released in June 2020 failed to report the death of a Petrobras civil servant, a member of the Internal Accident Prevention Committee (CIPA) who was working closely with managers to mitigate risks in the RPBC control room, as well as two other deaths (Amazonas facility): one of a civil servant and other of an outsourced worker, none included in the bulletin, according to a Federal Nacional dos Petroleiros report.^27,28^

The number of civil servants (46,000) informed to the MME does not match the actual number of employees at Petrobras, as it is being reduced as a result of voluntary termination, with no replacement of retiring employees, as stated in a report.^13^

### VOLUNTARY TERMINATION

In 2020, Petrobras had four voluntary terminations, three launched in 2019 for different types of employees (retirees, employees from areas in divestment process, and employees in corporate departments) and one in 2020. Of the 10,567 employees who joined the four voluntary termination throughout 2019 and 2020, 4,815 left the company between January and December 2020.^13^

Therefore, it is important to highlight the inconsistent data comparison with the number of employees Petrobras has provided to the MME because, based on the same rationale, we can assume that the data on COVID-19 released to the MME related to suspected and confirmed cases and deaths due to COVID-19 are probably unreliable.

In this context, it is interesting to consider the importance of professionals to engage in a set of actions to actively search for cases of COVID-19, to analyze information from various sources - from the MS to those trade unions and industry federations, press digital media collected - to compare and check the number of cases on these platforms with the overall number of cases.

In a timeline, after searching for news on digital media using terms such as “surto COVID-19 petroleiros” (COVID-19 outbreak oil and gas workers) and “surto COVID-19 Petrobras” (COVID-19 outbreak Petrobras), we found a significant difference between what was reported in the press and the data official sources reported on the health of oil and gas workers during the pandemic.

In April 2020, an estimated total of 1,124 cases of workers with COVID-19 symptoms were reported on rigs in Espírito Santo, Santos Basin, and Urucu (Manaus), and 184 cases were confirmed and nine were hospitalized.

In May 2020, 112 confirmed and 101 suspected cases were reported in Petrobras production facilities in Campos Basin. In Betim (Regap), 39 confirmed cases and 3 deaths were reported, and 3,747 cases were notified. In Ceará, a massive contamination was observed, with 42 confirmed positive cases. A total of 16 oil and gas workers were diagnosed with COVID-19 at the Polo Industrial de Guamaré, in Rio Grande do Norte. A refinery located in Cubatão, São Paulo reported three deaths and another six positive cases in Espírito Santo. Notably, all of these cases were not reported in the MME bulletins.

These underreported cases and fail to report official data on oil and gas workers raise several questions to be addressed on occupational health issues during the COVID-19 pandemic, as well as the preexisting situations.

The transmissibility of SARS-CoV-2 in the various settings of oil and gas industry becomes a challenging issue for decision-maker managers to provide adequate labor conditions and safety for workers, in view of the high incidence of confirmed cases of COVID-19, deaths, and sick leave, both among Petrobras civil servants and outsourced oil and gas workers.

Occupational illness is a reality in several industries due to the deterioration of working conditions, and it is up to managers to provide safer and more decent means for all workers to safeguard their health, and the risk of contamination with SARS-CoV-2 is a major challenge for professionals in the oil and gas industry and labor management.^29^

The SARS-CoV-2 pandemic has aggravated the inequalities of a conjuncture in which workers accumulate relevant losses of labor and social security rights. Therefore, the pandemic and associated health, economic, and social implications have deepened a context of intense labor fragility and deregulation.^30^

## FINAL CONSIDERATIONS

Numerous news published on digital media about cases, outbreaks, and deaths of oil and gas workers due to COVID-19 reveal the significantly unhealthy working conditions and prove that managers have failed to monitor workers’ health, with regard to the omission of official data and compulsory notifications on health and safety on oil and gas industry.

The underreporting and/or omission of information on workers’ health is of major concern to trade unions and labor rights watchdogs. Managers should be responsible for ensuring the necessary labor conditions for workers to minimize transmission and propose organizational measures in every labor activity in the oil and gas industry, to reduce the impact and incidence of COVID-19 cases and deaths.

It is worth noting that approaching workers to identify their demands, including health problems, cases of compulsory notification and/or unhealthy working conditions, is of paramount importance to map the actual needs of these workers, from an epidemiological surveillance perspective, to investigate the health of oil and gas workers, to gather information on COVID-19 cases and to early identify them and adequately address different control measures in an industry the pandemic has greatly affected.

As a result of COVID-19 infections in countless workplaces in Brazil oil and gas industry, it is increasingly necessary to foster studies, recommendations, and scientific opinions to support investigations on the causal link between COVID-19 and work, as well as further debates on improvement, preventive measures to mitigate workers’ exposure to hazardous conditions and SARS-CoV-2 transmission and new variants and difficulties in facing the pandemic in occupational health.
